# Emodin-8-*O*-β-d-Glucoside from *Polygonum Amplexicaule D. Don var. Sinense* Forb. Promotes Proliferation and Differentiation of Osteoblastic MC3T3-E1 Cells

**DOI:** 10.3390/molecules16010728

**Published:** 2011-01-18

**Authors:** Mei-Xian Xiang, Zong Xu, Han-Wen Su, Jinyue Hu, Yun-Jun Yan

**Affiliations:** 1 College of Life Science and Technology, Huazhong University of Science and Technology, Wuhan 430074, China; 2 College of Pharmacy, South-Central University for Nationalities, Wuhan 430075, China; 3 Renmin Hospital, Wuhan University, Wuhan 430060, China

**Keywords:** emodin-8-O-β-d-glucoside, *Polygonum amplexicaule D. Don var. sinense* Forb. (Polygonaceae), proliferation, differentiation, osteoblastic MC3T3-E1 cell

## Abstract

*Polygonum amplexicaule* D. Don *var. sinense* For*b.* (Polygonaceae) (PAF) is a famous traditional herb used to treat fractures, rheumatoid arthritis, muscle injury and pain. The present study was designed to investigate a PAF derived-chemical compound emodin-8-*O*-β-d-glucoside (EG) on the proliferation and differentiation of osteoblastic MC3T3-E1 cell *in vitro*. A compound was isolated from PAF extract by HPLC and identified as emodin-8-*O*-β-d-glucoside (EG) by spectroscopic methods. EG significantly promoted cell proliferation at 0.1–100 ng/mL, and increased the cell proportion in S-phase from 16.34% to 32.16%. Moreover, EG increased alkaline phosphatase (ALP) expression in MC3T3-E1 cells at the concentration from 0.1 to 100 ng/mL and inhibited PGE_2_ production induced by TNF-α in osteoblasts at the concentrations ranging from 10–100 ng/mL, suggesting that cell differentiation was induced in MC3T3-E1 osteoblasts. Taken together, these results indicated compound EG directly stimulated cell proliferation and differentiation of osteoblasts, therefore this study preliminarily explored the pharmacological mechanism of PAF to promote the healing of bone rheumatism and various fractures.

## 1. Introduction

In recent years, Traditional Chinese Medicines (TCMs) have become important sources of potential therapeutic agents because of their tremendous diversity. Various strategies have been used to investigate the phytochemical and pharmacological effects of TCMs based on their therapeutic potentials [1]. *Polygonum amplexicaule D. Don var. sinense Forb.* (Polygonaceae) (PAF) is a herbal drug used to treat fractures, rheumatism, osteoporosis, muscle injuries and pain. It has also been reported to be effective to treat atherosclerosis and its antibiotic and antivirus effects were also examined with positive results. However, which chemical compound in PAF plays the therapeutic role is not clear, and its pharmacological mechanism to promote the healing of fractures is still unknown.

The chemical compounds from herbal medicines have fewer side effects and are more suitable for long-term use compared with total extracts. In this study, the chemical compound emodin-8-*O*-β-d-glucoside (EG) was isolated from PAF. EG is also the component in traditional Chinese medicinal herb *Polygonum cuspidatum* Sieb. et Zucc and functions to protect from focal cerebral injury induced by ischemia and reperfusion [2]. However, no results have been reported about its potential to promote the healing of bone injury.

The MC3T3-E1 osteoblastic cell line is a widely adopted model for the research of osteogenesis *in vitro* [3]. By use of MC3T3-E1 cell model, several medical herbs such as *Icariin* [4], *Actaea racemosa* [5], *safflower seeds* [6], *Ulmus davidiana planch* [7], *Drynaria fortunei* [8], *Drynariae Rhizoma* [9], *Soybean isoflavones* [10,11] and *Homalomen occulta* [12] have been proven to be functional to promote osteogenesis. The purpose of this study is to identify the chemical compound in PAF promoting the healing of bone fractures, which may be able to replace PAF in clinical use.

## 2. Result and Discussion

### 2.1. Indentification of PAF Compounds

**Figure 1:**
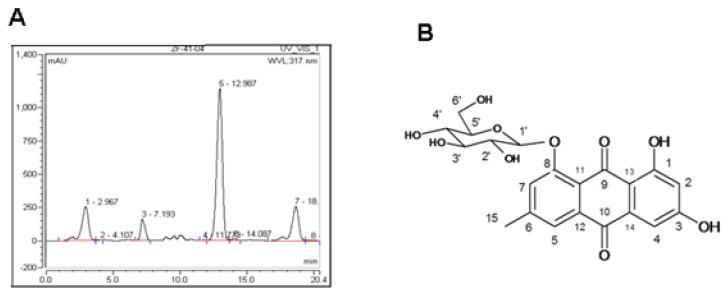
HPLC and the structure of emodin-8-*O*-β-d-glucoside. (A) HPLC chromatogram. PAF extract components were separated by semi-preparative HPLC and eluted with methyl alcohol/water (55:45); (B) The structure of emodin-8-*O*-β-d-glucoside.

As PAF extract promotes cell proliferation (data not shown), we isolated the components in PAF extract by HPLC (Figure 1A), and a compound in the absorption peak with a retention time of 13.0 min was harvested for further spectroscopic analysis.

By comparison of the spectroscopic parameters with published data [13], this compound was identified as emodin-8-*O*-β-d-glucoside (EG) (Figure 1B).

### 2.2. The Effect of EG on Proliferation of MC3T3-E1 Cells

To elucidate whether EG plays a role in PAF induced cell proliferation, MC3T3-E1 cells were treated with 1 ng/mL EG for 7 days. Cell numbers were detected at day 1 to day 7 by CCK-8 assay. The results showed that EG significantly promote cell proliferation from day 1 to day 7. The dose-response curves indicated that 0.01 to 100 ng/mL of EG significantly stimulated the proliferation of MC3T3-E1 cells (*p* < 0.01). Thus, we can conclude that EG contributed to PAF induced cell proliferation.

### 2.3. The Effect of EG on Cell Cycle Progression

**Figure 3:**
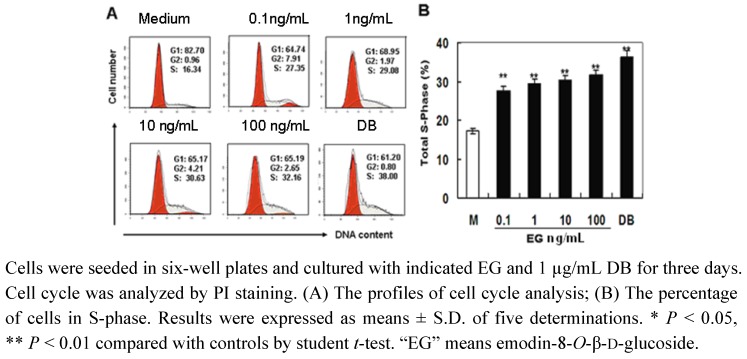
EG promotes cell cycle progression in MC3T3-E1 cells.

Cell proliferation is regulated by cell cycle progression. MC3T3-E1 cells were treated with various concentrations of EG, and cell cycle analysis was performed by PI staining and flow cytometry. The results showed that EG dose-dependently up-regulated the cell proportion in S-phase significantly (Figures 3A and 3B). When cells were cultured with 0.1 to 100 ng/mL of EG, the cell proportion in S-phase increased from 16.34% to 32.16% (Figure 3A and 3B). These results indicated that EG promoted cell proliferation by regulating cell cycle progression.

### 2.4. The Effect of EG on ALP Expression in MC3T3-E1 Cells

**Figure 4:**
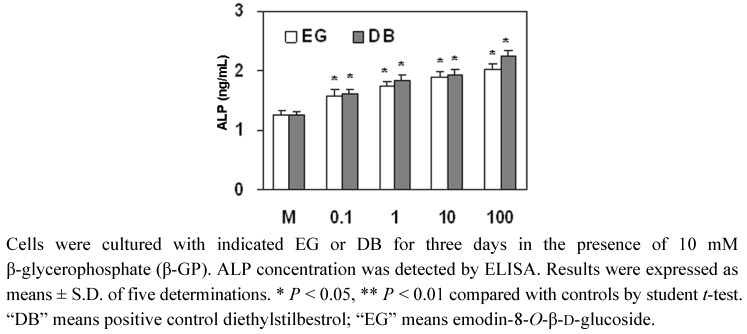
The effect of EG on ALP expression in MC3T3-E1 cells.

The healing of fractures is related to the differentiation of osteoblasts. In this study, the effect of EG on the expression of ALP, a marker for osteoblast differentiation, was detected in MC3T3-E1 cell. The results showed that the treatment of MC3T3-E1 cells with 0.1 to 100 ng/mL of EG significantly up-regulated the expression of ALP (Figure 4), which was similar to that of the positive control diethylstilbestrol (DB). Therefore, the herb PAF has a markedly positive effect on the differentiation of MC3T3-E1 cells. 

### 2.5. The Effect of EG on PGE2 Production in MC3T3-E1 Cells

**Figure 5:**
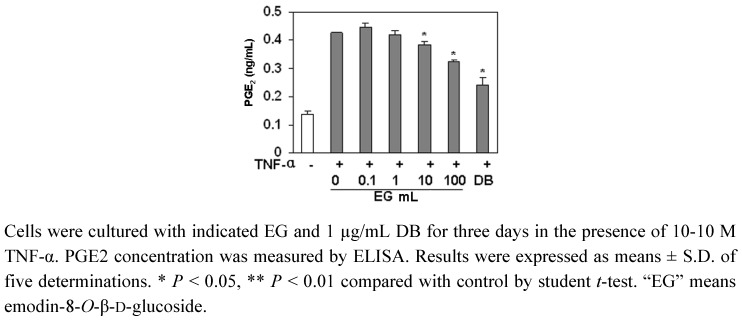
The effect of EG on PGE_2_ production in MC3T3-E1 cells.

PGE_2_ is an endogenous factor to stimulate osteolysis through the EP4 receptor [14]. We examined the effect of EG on PGE_2_ production in MC3T3-E1 cells. As shown in Figure 5, the treatment of MC3T3-E1 cells with TNF-α increased the expression of PGE_2_, but EG (10–100 ng/mL) significantly inhibited the expression of PGE_2_ induced by TNF-α.

To investigate the pharmacological mechanism of PAF to promote the healing of fractures and rheumatism, we selected the well-recognized osteoblastic MC3T3-E1 cell line as *in vitro* model. As the healing of fractures and rheumatism is related to the proliferation and the differentiation of osteoblastic cell, we examined cell growth in osteoblastic MC3T3-E1 cells by CCK-8 assay *in vitro*. The results showed that the low dosage of EG stimulated cell growth. At the same time, the results of cell cycle assay demonstrated that EG effectively promoted cell cycle progression, the proportion of cells in S-phase of cells significantly increased. These results indicated that EG from PAF promoted cell proliferation by regulating cell cycle progression. Moreover, the effect of EG on cell growth in osteoblastic MC3T3-E1 cells was comparable with DB (as positive control).

ALP is the most widely recognized biochemical marker for osteoblastic activity [15]. Although its precise mechanism of action is poorly understood, this enzyme is believed to play an important role in bone metabolism [16]. Therefore, we examined the effects of EG from the root tubers of PAF on the ALP activity of osteoblastic MC3T3-E1 cells. The results showed that the compound EG from PAF increased ALP expression, and the effect of EG on ALP expression was similar to that of the positive control DB.

PGE_2_ is an endogenous factor to stimulate osteolysis through the EP4 receptor [14]. It is known that some bone-resorbing agents like TNF-α [17,18,19] act on osteoblasts and stimulate PGE_2_ release from osteoblasts [20]. The released PGE_2_ acts on stroma cells and enhances factors that support the differentiation from stem cells to osteoclasts to augment osteolysis. Furthermore, it is known that the presence of PGE_2_ caused a significant decrease in bone ALP activity and a corresponding increase in bone acid phosphatase activity. In the present study, EG inhibited PGE_2_ production induced by TNF-α in osteoblasts, suggesting that EG may inhibit the osteolysis by down-regulating the release of PGE_2_ induced by TNF-α. This result partly elucidated the pharmacological mechanism of PAF to promote the healing of bone fractures and rheumatism.

## 3. Experimental

### 3.1. Materials

MC3T3-E1 cell line was provided by the Institute of Biochemistry and Cell Biology, Shanghai Institutes for Biological Sciences, Chinese Academy of Sciences (Shanghai, China). Tissue culture media and reagents, fetal bovine serum (FBS) were from Hyclone INC. (Logan City, Utah, USA). Diethylstilbestrol was from Shanghai Sine Kangjie Pharmaeutical CO. Ltd. (Shanghai, China). All other reagents were of analytical grade and purchased from China National Pharmaceutical Industry Corporation. Ltd. (Shanghai, China).

### 3.2. Plant Material 

The fresh root tubers of PAF were collected in Enshi County of Hubei Province, China, in October 2008 and authenticated by the College of Pharmacy, South Central University for Nationalities (SCUN). The voucher specimen (SCUN0810) was deposited in the Herbarium of the College of Pharmacy, SCUN, China. 

### 3.3. Extraction and Isolation Procedures

The dried root tubers of PAF (10 kg) were extracted with 95% EtOH (25 L × 3) and then successively partitioned with petroleum ether (P.E.) (3.0 L × 3), EtOAc (3.0 L × 3) and *n*-BuOH(3.0 L × 3). The combined EtOAc extract (490 g) was chromatographed on silica gel (200-300 mesh, China) with cyclohexane-acetone (9:1, 8:2, 7:3, 1:1, 3:7, 0:1, *v/v*) to give seven fractions (fr.1-fr.7). Fraction 6 (11 g) was separated on a RP-silica gel column (50 μm, 2.4 × 20 cm, YMC Co. Japan) and eluted with MeOH-H_2_O (1:9, 2:8, 3:7……9:1 500 mL/gradient) to give seven fractions (Fr F_a_ to F_g_). Fr F_e_ (130 mg) was separated by Dionex semipreparative HPLC and eluted with MeOH-H_2_O 55:45. Chromatography conditions: chromatography column Cosmosil 5C18-MS-II 10 × 250 mm (Nacalai tesque, Japan), wavelength set at 317 nm, at the temperature of 30 °C and at the flow rate of 1.5 mL/min. The retention time of EG was 13.0 min. 85.7 mg of compound was obtained (yield 0.857%).

### 3.4. Sample Preparation

Ten mg of emodin-8-*O*-β-d-glucoside (EG) and diethylstilbestrol (DB, as positive control) were weighed respectively, and then diluted with DMEM medium. The concentrations of DB and EG ranged from 0.01–100 ng/mL.

### 3.5. Cell Culture

MC3T3-E1 cells were cultured in DMEM supplemented with 10% FBS, 100 μg/mL penicillin and 100 U/mL streptomycin in a mixture of 95% air and 5% CO_2_ in a humidified incubator at 37 °C.

### 3.6. Cell Proliferative CCK-8 Assay

The cells (2 × 10^4^ cells/well) were seeded in 96-well plates and cultured for 24 h to obtain adherent monolayer cells. After washed twice with phosphate-buffered saline (PBS), the cells were treated with various concentrations of PAF extracts or EG for 1–7 days, and cell proliferation was evaluated by cell count kit-8 assay (CCK-8, Beyotime Inst Biotech., China) according to the manufacturer’s instructions [21]. Briefly, 10 μL CCK-8 was added to each well (100 μL medium). After incubation for 2 h at 37 °C, the absorbance at 450 nm was measured by an enzyme-linked immunosorbent assay (ELISA) plate reader. The experiments were carried out in five replicates and the results were expressed as mean ± S.D.

### 3.7. Cell Cycle Analysis

The cells (2 × 10^5^ cells/well) were seeded in 6-well plates and cultured for 24 h to obtain adherent monolayer cells. The adherent cells were cultured for another 24 h in serum-free medium for synchronization. After washing twice with PBS, the cells were cultured with various concentrations of PAF extracts or EG. Then the cells were harvested and fixed with 70% cold ethanol at 4 °C overnight. After washing with PBS and centrifuging at 2,000 rpm for 3 min, the cells were incubated with 100 μL RNase (1 mg/mL) for 30 min at 37 °C, and then 50 μL (1 mg/mL) propidium iodide (PI, Jingmei, Shenzhen, China) was added and the cells were incubated at 4 °C for 30 min in the dark. The cell cycle was detected by flow cytomerty [22,23] (BD Bioscience, San Jose, CA, USA). The data were analyzed by Modfit software (Verity Software House, Topsham, ME, USA).

### 3.8. Alkaline Phosphatase (ALP) Activity Assay

MC3T3-E1 cells were cultured to grow to 80–100% confluent. The medium was replaced with phenol red-free α-MEM containing 5% charcoal-dextran-treated FBS (Cd-FBS; GIBCO). Cells were cultured with various concentrations of EG, supplemented with 10 mM β-glycerophosphate (β-GP) (G9422, Sigma Chemical Co., St. Louis, MO), which was added to initiate *in vitro* cells differentiation [24,25]. After 3 days, the medium was removed and the monolayer cells were gently washed twice with PBS. The cells were harvested to 1.5 mL tubes and centrifuged for 20 min at 3,000 rpm. Supernatant was discarded, and PBS (PH 7.2–7.4) was added to prepare a solution with 1 × 10^7^ cells/mL. Freeze-thaw cycles were repeated three times to break the membrane of the cells to release intracellular components. Then the solution was centrifuged for 15 min at 12,000 rpm. The supernatant was harvested for the measurement of ALP concentration by use of an ELISA kit (R&D System Inc., Minneapolis, MN, USA). The experiments were carried out in five replicates and data were expressed as mean ± S.D.

### 3.9. Prostaglandin (PG) E2 Assay

The cells were cultured according to the method as mentioned above except that the β-GP was replaced with 10^−10^ M TNF-α. The content of PGE_2_ in the supernatant was measured with an ELISA kit (R&D System Inc., Minneapolis, MN, USA) according to the manufacturer’s instructions [26]. Immediately after the color development, the absorbance is read at 450 nm. The experiment was carried out in five replicates and data were expressed as mean ± S.D.

### 3.10. Statistics

All experimental results were performed at least three replicates. Results were expressed as mean plus or minus standard deviation (SD). Differences between groups were examined for statistical significance by student’s *t*-test, and *P* < 0.05 was considered statistically significant.

## 4. Conclusions

These results indicate that the compound EG directly stimulated cell proliferation and differentiation of osteoblasts, therefore this study preliminarily explored the pharmacological mechanism of PAF to promote the healing of bone rheumatism and various fractures.

## Figures and Tables

**Figure 2:**
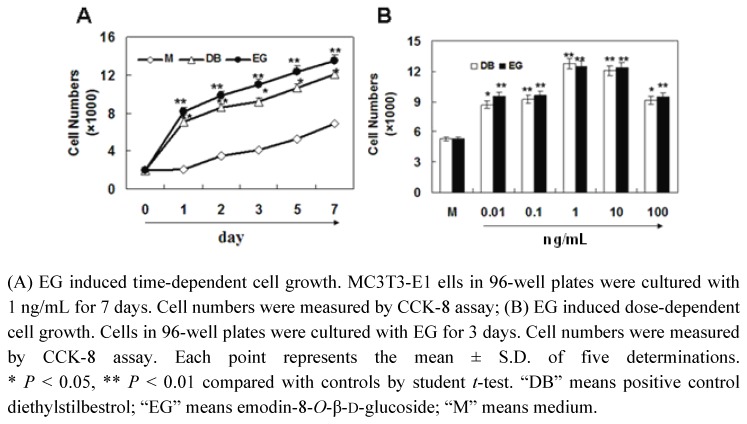
EG increases the proliferation of MC3T3-E1 cells.
